# Observations on morphologic and genetic diversity in populations of *Filoboletus manipularis* (Fungi: Mycenaceae) in southern Viet Nam

**DOI:** 10.1080/21501203.2014.902402

**Published:** 2014-04-04

**Authors:** Galina A. Vydryakova, Olga V. Morozova, Scott A. Redhead, John Bissett

**Affiliations:** a ECORC, Agriculture and Agri-Food Canada, Ottawa, Canada; b Laboratory of Systematics and Geography of Fungi, Komarov Botanical Institute RAS, Saint Petersburg, Russia; c Vietnam-Russian Tropical Research and Technological Centre, Southern Branch, Ho Chi Minh City, Viet Nam

**Keywords:** *Filoboletus manipularis*, morphology, population genetics, basidiomycete mating system, multinucleate chimeric basidiomata

## Abstract

The morphological variation of basidiomata of *Filoboletus manipularis* (Berk) Singer collected in southern Viet Nam was studied. Phylogenetic analyses comprising three gene loci indicated that these collections, although exhibiting widely varying morphologies, represented a single species with a population composed of genetically diverse, sexually compatible monokaryon parental strains. No correlation was found between any aspect of morphological variation and intraspecific phylogenetic patterns for the three gene loci studied. Primers were designed to amplify the intron-rich 5′ region of the translation elongation factor 1-α gene (*tef1α*) and amplicons cloned and sequenced to characterize the parental haplotypes for individual basidiomata. The presence of recombination over the entire morphological diversity seen was confirmed by split decomposition analysis and analysis of gene diversity indicated a lack of allelic fixation within local populations. On several occasions, more than two apparent parental haplotypes were characterized from individual basidiomata, indicating that at least some basidiomata are chimeric or otherwise develop from a multinucleate condition. The literature supporting our observations of the occurrence of multinucleate basidiomata is reviewed and possible mechanisms for this phenomenon are proposed.

## Introduction

*Filoboletus manipularis* (Berk.) Singer (≡*Favolaschia manipularis* (Berk.) Teng) is a luminescent fungal species widely distributed on rotting wood throughout Asian, Australasian and Pacific tropical regions (Corner 1954; Pegler 1986; Manimohan and Leelavathy 1989; Liu and Yang 1994; Desjardin et al. 2008). Fifteen species are currently accepted in the genus *Filoboletus* (http://www.mycobank.org); all described prior to 1996, with species circumscribed on the basis of morphological and anatomical characteristics (e.g. Liu and Yang 1994; Maas Geesteranus and Horak 1995). The phylogenetic relationships among species accommodated in *Filoboletus* have not been studied, and even their generic affinities remain largely unresolved. Mycobank (http://www.mycobank.org/) gives *Favolaschia* as the currently accepted generic placement for the species. However, phylogenetic analysis of available sequences (not shown) place *F. manipularis* within a *Mycena* clade closest to *M. rubreomarginata* and distant from *Favolaschia* species. According to Desjardins et al. (2008), *F. manipularis* and related species currently assigned to *Filoboletus* and *Poromycena* require a new generic name since none are closely related to the type species of these genera. Publically available molecular data for *Filoboletus* is limited to five Genbank sequences that are fragments either of the 28S ribosomal DNA gene (*Poromycena* sp. AF261421, *F. gracilis* AF261422, *Poromycena manipularis* AF261423; Moncalvo et al. 2002) or internal transcribed spacer 2 (*F. manipularis* AB509828, *F*. aff. *manipularis* AB509539). Therefore, the current study was undertaken to determine if the morphological variation observed in *F. manipularis* represents more than one species or could be correlated to the infraspecific phylogenetics of the species. Collections of this species taken from southern Viet Nam in the current study exhibited a wide range of variation in morphologies, similar to morphological observations from previous studies of the species from diverse tropical regions (Corner 1954). DNA was isolated from individual basidiomata representing the diverse morphology observed and their genetic relationships determined from phylogenetic analyses of three gene loci.

## Methods

### Collections

Individual and clustered basidiomata identified as *F. manipularis* were collected in Cát Tiên National Park and Dồng Nai Culture and Nature Reserve in southern Viet Nam (Table 1). These two reserves are core zones of the Dồng Nai Biosphere Reserve and together comprise 172,223 hectares of water surface (Trị An reservoir), seasonally flooding grasslands and semi-evergreen and deciduous lowland tropical forests located in Dồng Nai, Lâm Dồng and Bình Phước provinces in Viet Nam. Collections were taken in June 2010, during the rainy season (average annual temperature and precipitation 26°C and 2400 mm, respectively), in three separate regions of the reserves, near Nam Cát Tiên, Bàu Sấu and Mã Dà-Vïnh Cửu, located approximately 15–50 km distant from one another and representative of the habitat diversity of the forested portion of the reserves. All of the fruit bodies were found on the forest floor on rotting wood of undetermined origin, mostly in forests dominated either by *Lagerstroemia calyculata* Kurz or *Dipterocarpus dyeri* Pierre. Collected fruit bodies were air-dried on location and the separate collections transported for subsequent study in sealed plastic bags containing silica gel. Observations on the color (according to Kornerup and Wanscher 1978) and macromor-phology of basidiomata were recorded on fresh specimens in the field, and microscopic observations performed on the air-dried specimens as described previously (Morozova et al. 2012) after transport to the investigating laboratory in Saint Petersburg, Russia. All specimens are deposited in the Komorov Botanical Institute Herbarium, Leningrad (LE).

**Table 1.** Collections of *F. manipularis* studied.

				Genbank no.		
Basidiome	Collection no.^a^	Date of collection	Locality	ITS	*rpb2*	*tef1α*^b^
Gl	LE254356	8 June 2010	Nam Cát Tiên	KF746989	KF746986	KF746965 KF746964 (6)
						(5)
G2	LE 253916	8 June 2010	Nam Cát Tiên	KF746998	KF746986	KF746972 (9)
						KF746971 (4)
G3	LE 254357	21 June 2010	Mã Dà-Vïnh Cửu	KF746994	KF746986	KF746980 (7)
						KF746978 (2)
						KF746979 (2)
G6	LE 254358	8 June 2010	Nam Cát Tiên	KF746995	KF746987	KF746982 (9)
						KF746981 (2)
G7	LE 253912	15 June 2010	Nam Cát Tiên	KF746997	KF746986	KF746983 (19)
					KF746996	KF746984 (4)
G9	LE 254359	8 June 2010	Nam Cát Tiên	KF746992	KF746987	KF746985 (9)
G11	LE 254360	12 June 2010	Bàu Sấu	KF746991	KF746987	KF746962 (2)
						KF746963 (2)
G24	LE 253914	4 June 2010	Nam Cát Tiên	KF746988		KF746967 (6)
						KF746966 (3)
G25	LE 253916	15 June 2010	Bàu Sấu	KF746990	KF746987	KF746969 (7)
						KF746970 (7)
						KF746968 (2)
G30	LE 253913	15 June 2010	Nam Cát Tiên	KF746993	KF746986	KF746975 (8)
						KF746974 (5)
						KF746973 (3)
G31	LE 253915 Sato et al.^c^	4 June 2010	Nam Cát Tiên Yakushima Island, Japan	AB509828		KF746977 (5)
						KF746976 (2)
	Sato et al.^c^		Yakushima Island, Japan	AB509828		

Notes: ^a^LE – mycological herbarium of the Komarov Botanical Institute, RAS, St Petersburg.

b in brackets – number of identical *tef1α* clone sequences.

c Sato H, Tsujino R, Kurita K, Yokoyama K, Agata K, ‘Use of ITS sequences for resolving species richness, ecological differentiation and geographical distribution of macrofungi in Yakushima Island, Japan', Genbank accession AB509828, 19 June 2009.

### DNA isolation and sequencing

Fruit body tissues were extracted in an aseptic manner from the pileus of individual basidiomata. Genomic DNA was isolated from the harvested tissues of each single basidiomata using the E.Z.N.A Fungal DNA Mini Kit (OMEGA Bio-Tek, Norcross, GA, USA) according to the manufacturer's protocol. The following genetic loci were amplified using the primers and amplification protocols listed in Table 2: (a) a region of nuclear rDNA containing the internal transcribed spacer regions 1 and 2 (ITS 1–2) and the 5.8S rRNA gene, (b) a fragment of the second largest subunit of the RNA polymerase II gene (*rpb2*) comprising approximately 250 bp and (c) a fragment at the 5′ end of the translation elongation factor 1-α gene (*tef1α*, eEFla) approximately 850 bp containing four introns.

**Table 2.** PCR primers and protocols.

Locus	Primers	Amplification protocol	Reference
ITS	ITS 1-F (5′-CTTGGTCATTTAGAGOAAGTAA-3′)	3 min at 95°C; 35 cycles of (30 sec at 95°C, 30 sec at 55°C, 1 min at 72°C); 10 min at 72°C	Gardes and Bruns (1993)
	ITS4-B (5′-CAGGAGACTTGTACACGGTCCAG-3′)		
*rpb2*	RPB2-filoF (5′-ATTCCCCGACCATAACCAG-3′)	3 min at 94°C; 40 cycles of (1 min at 94°C, 1 min at 65°C, 1 min at 72°C); 10 min at 72°C	Current study
	RPB2-filoR (5′-ACACAGGATGGCGACGATAG-3′)		
*tef1α*	TEF-filoF (5′-TTTCTTGCAGCGCTTGTTCT-3′)	30 sec at 98°C; 35 cycles of (10 sec at 98°C, 30 sec at 65°C, 30 sec at 72°C); 10 min at 72°C	Current study
	TEF-filoR (5 ‘-GTGCCAATACCACCGATCTT-3 ‘)		

For ITS and *rpb2*, amplifications were performed using a Labnet Multigene thermocycler (Labnet Intl., Edison, NJ, USA). PCR reactions were prepared in 10 µL volume containing the following mix: 1 µL 10X Titanium Taq Buffer (Clontech, Mountain View, CA, USA), 0.5 µL 2 mM dNTP, 0.32 µL each of 5 µM upper and lower primers, 7.76 µL sterile distilled water and 0.1 µL Titanium Taq Polymerase (Clontech). For *tef1α*, amplifications were performed using a Biometra TProfessional thermocycler (Biometra GmbH, Goettingen, Germany) with the Phusion Hot Start II High-Fidelity DNA Polymerase according to manufacturer's protocol (Finnzymes, Espoo, Finland).

Sequencing reactions were prepared using the ABI Prism® BigDye™ Terminator reaction kit (v3.1, Applied Biosystems Inc., Foster City, CA, USA) in 10 µL volume and 1/8 dilution using 5X sequencing buffer. The cycle sequencing reaction contained the following mix: 1.75 µL 5X Sequencing Buffer, 0.5 µL BigDye V3.1 Mix, 0.5 µL of 3.2 (iM primer, 6.25 µL sterile distilled water, 1.0 µL (10–40 ng) PCR template, and employing the following amplification protocol: 25 cycles each of 30-sec denaturation at 96°C, 15 sec annealing at 50°C and 4 min extension at 60°C. Primers used for PCR were also used for sequencing. Sequences were obtained using an ABI Prism 3100 Genetic Analyzer (Applied Biosystems).

Cloning was performed on amplicons arising from amplification of *tef1α* using the TOPO TA Cloning Kit (Invitrogen, Carlsbad, CA, USA) following the manufacturer's protocol. PCR for *tef1α* was performed using a high-fidelity polymerase to reduce the frequency of cloning errors during nucleotide transcription. Nevertheless, 12 singleton *tef1α* clone sequences were excluded from phylogenetic analyses and only clone sequences obtained two or more times from individual basidiomata were included to reduce the possibility or frequency of cloning errors. Genbank accession numbers for the sequences are provided in Table 1.

### Phylogenetic analysis

DNA sequences were aligned using the multiple sequence alignment program MAFFT and G-INS-i strategy (http://mafft.cbrc.jp/alignment/server/), or the FFT-NS-i strategy for *tef1α* fragments containing introns. The coding region and introns in the *tef1α* locus were located from an alignment with annotated sequences of *Mycena plumbea* (Genbank GU187729) and *Phyllotopsis* sp. (DQ059047) and introns were removed for analyses of the *tef1α* locus that included outgroup taxa. Parsimony phylogenetic analyses to visualize the degree of genetic variation for all three loci were performed with PAUP 4.0b. 10 (Sinauer Associates, Inc., Sunderland, MA, USA; Swofford 1993), using a heuristic search with a starting tree obtained via stepwise addition, 200 Maxtrees and otherwise default parameters. Bayesian inferences were used for phylogenetic analyses of the *tef1α* region. Sequences containing introns and with the intron regions deleted were analyzed separately, the latter analysis including *Mycena plumbea* (GU 187729) as the phylogenetically closest available outgroup representative determined from BLAST comparison of GenBank sequences. Nucleotide substitution models were determined using MrModeltest 2.3 (Nylander 2004; http://www.softpedia.com/get/Science-CAD/MrModeltest.shtml). The unconstrained substitution model (GTR + I + G) was selected by both Akaike and Bayesian Information Criteria and applied in all analyses of *tef1α* fragments. Bayesian estimations of phylogeny based on Metropolis-coupled Markov chain Monte Carlo sampling were performed using MrBayes v.3.2.1 (http://mrbayes.sourceforge.net/), with two simultaneous runs of four incrementally heated chains running for ten million generations. Posterior probabilities were obtained from 50% majority rule consensus trees sampled every 5000 generations. Probability values for posterior probabilities greater than 90% are shown on the phylograms. Patterns of recombination were visualized by split decomposition in SplitsTree v.4.12.3 (Huson and Bryant 2006; http://www.splitstree.org), using pairwise distances with the Kimura K3ST model. The presence or absence of recombination was determined using the pairwise homoplasy index (PHI) implemented in SplitsTree. The level of heterozygosity among individuals within populations (localities) and among populations was determined from the *tef1α* exon data using the program FSTAT to analyze gene diversity (http://www2.unil.ch/popgen/softwares/fstat.htm).

## Results

### Morphological observations

**Figures 1–6.**
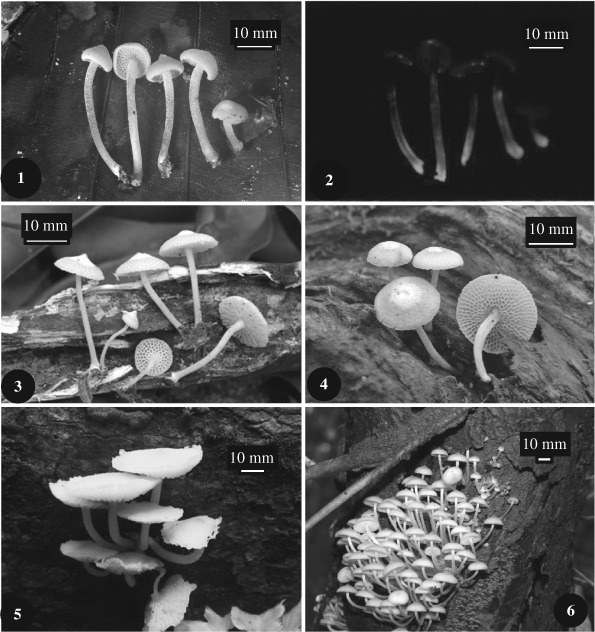
*Filoboletus manipularis* from southern Viet Nam showing variation in basidioma gross morphology. Figure 1: LE 254357 (G3), white basidiomata of medium size and conical-umbonate pilei, photograph A.E. Kovalenko. Figure 2: LE 254357 (G3) showing stipes luminescent from below, photograph A.E. Kovalenko. Figure 3: LE 253914 (G24) white basidiomata with umbonate pileus, photograph O.V. Morozava. Figure 4: LE 253917 (G22) pink basidiomata with rounded pileus, photograph O.V. Morozava. Figure 5: LE 253916 (G2) large, pink basidiomata with plane or depressed pileus. Figure 6: LE 253912 (G7) cluster of cream to beige colored caespitose basidiomata.

Considerable variation was seen in the size, shape, color and luminosity of basidiomata (Table 3, Figures 1–6), although fruiting bodies arising within a single cluster were relatively uniform in morphology. At maturity fruiting bodies varied from 0.5 to 6.0 cm in pileus diameter with stipes 2.0 to 7.0 cm in length. The pileus could be conical, rounded, plane or depressed and umbonate or not. Color at maturity varied from white to cream or beige (near 4A2 ‘yellowish white', Kornerup and Wanscher 1978) or pale pink (6A2 ‘orange white'). Developing basidiomata initially could be white or brownish (5C4 ‘brownish orange') with the intensity of brown pigment diminishing with maturity. There were conspicuous differences in luminosity patterns with only the cap luminescent or luminescent from underneath, only the stipe luminous, the entire fruiting body luminescent, or showing no luminescence throughout.

**Table 3.** Morphological observations on *F. manipularis* basidiomes.

Basidiome	Size^a^	Color^b^	Shape	Luminosity^c^	Basidiopores	Cheilocystidia	Pileocystidia
Gl	**L**	**W**	Plano-convex	C (underneath)	5.8–7.6 (8.6) × 4.2–5.5 µm, *Q* = 1.3–1.8 (2.0)	Lageniform to fusiform sometimes slightly diverticulate in the apex	Pileocystidia and excrescences of hyphae simple, small and rare
G2	**L**	**P**	Piano-depressed	S	6.5–8.6 × 4.0–5.3 µm, *Q* = 1.5–1.9	Lageniform or irregularly shaped, without excrescences	Pileocystidia and excrescences of hyphae small and rare
G3	**S**	**W**	Umbonate	**S**	6.0–7.6 × 4.5–5.0 µm, *Q* = 1.4–1.6	Lageniform to fusiform without excrescences	Pileocystidia and excrescences of hyphae abundant
G6	**S**	**W**	Convex	**C** (underneath)	6.2–8.4 × 4.2–5.6 µm, *Q* = 1.3–1.7	Lageniform, sometimes slightly diverticulate in the apex	Pileocystidia and excrescences of hyphae simple, small and rare
G7	**S**	**W**	Umbonate	**S** (lower part)	6.4–8.3(9.0) × 4.5–5.5 µm, *Q* = 1.3–1.8	Lageniform, sometimes slightly diverticulate in the apex	Pileocystidia rare, terminal cells of hyphae with excrescences
G9	**L**	**W**	Umbonate	A	6.2–8.6 × 4.2–5.3 µm, *Q* = 1.3–1.8	Lageniform to fusiform without excrescences	Pileocystidia not seen; excrescences of hyphae simple, small and rare
G11	**L**	**W**	Umbonate	**C**	na^d^	na^d^	na^d^
G24	**S**	**W**	Conico-umbonate	**S**	6.2–7.5 × 4.4–5.1 µm, *Q* = 1.3–1.6	Lageniform, often diverticulate in the apex	Pileocystidia rare, terminal cells of hyphae with excrescences
G25	**S**	**W**	Convex	**N**	6.5–7.3 × 4.9–5.3 µm, *Q* = 1.3–1.4	Irregularly shaped, diverticulate, with excrescences	Pileocystidia and excrescences of hyphae abundant
G30	**S**	**W**	Umbonate	**A**	6.0–8.5 × 4.3–5.4 µm, *Q* = 1.5–1.7	Lageniform, often diverticulate in the apex	Pileocystidia rare, terminal cells of hyphae with excrescences
G31	**L**	P	Depressed	**C**	6.5–9.0 × 4.2–5.5 µm, *Q* = 1.3–1.8	Lageniform, with long neck	Pileocystidia not seen, hyphae with rare excrescences

Notes: ^a^**S** – small, up to 2.5 cm diam., **L** – large, exceeding 2.5 cm diam.

b **W** – white to grayish or pale cream colored, **P** – pink.

c **C** – pileus, **S**- stipe, **A** – all luminous, **N** – nonluminous.

d na – not accessed.

Microscopic observations indicated a degree of correlation between the overall gross morphology and the microscopic anatomy of the basidiomata. In most instances larger basidiomata had noticeably larger basidia, shorter and mostly simple-lageniform cheilocystidia, much larger caulocystidia and pileocystidia absent or nearly so. Smaller basidiomata had cheilocystidia mostly diverticulate or irregularly branched, less ornate caulocystidia, and abundant, irregularly shaped pileocystidia. These differences allowed the differentiation of the two reasonably distinct morphologies described below.

***Filoboletus manipularis*** (Berk.) Singer, Lloydia, 8: 215, 1945 – *Favolus manipularis* Berk., Hooker's Journal of Botany and Kew Garden Miscellany, 6: 229, 1854; *Laschia caespitosa* var. *manipularis* (Berk.) Sacc, Sylloge Fungorum, 6: 407, 1888; *Laschia manipularis* (Berk.) Saca, Sylloge Fungorum, 6: 408, 1888; *Poromycena manipularis* (Berk.) R. Heim, Revue de Mycologie, 10: 35, 1945; *My cena manipularis* (Berk.) Métrod, Prodr. Fl. Mycol. de Madagascar, III, Les Mycènes, 87, 1949, f. 51 [non *M. manipularis* (Berk.) Saca, Sylloge Fungorum, 5: 272, 1887]; *Favolaschia manipularis* (Berk.) Teng, Cung-kuo Ti Chen-chun, [Fungi of China]: 760. 1963.

#### Morphology type 1 – Figure 7

Pileus 0.5–2.0 cm in diam., conico-campanulate to convex with conical umbo, hygrophanous, translucently reticulate, finely white pruinose, whitish, grayish to hyaline when moist, purely white to yellowish dried up. Hymenophore tubular, adnate or adnate-emarginate with a slightly decurrent tooth. Tubes 1.5–4 mm long, arranged in radial rows, numbering 5–7 in a row, with angular-round pores 0.5–1 mm wide, white. Stipe 20–60 × 0.5–2.5 mm, cylindrical, thickened in the base, hollow, white to hyaline, completely white pruinose. Odor indistinct.

**Figure 7.**
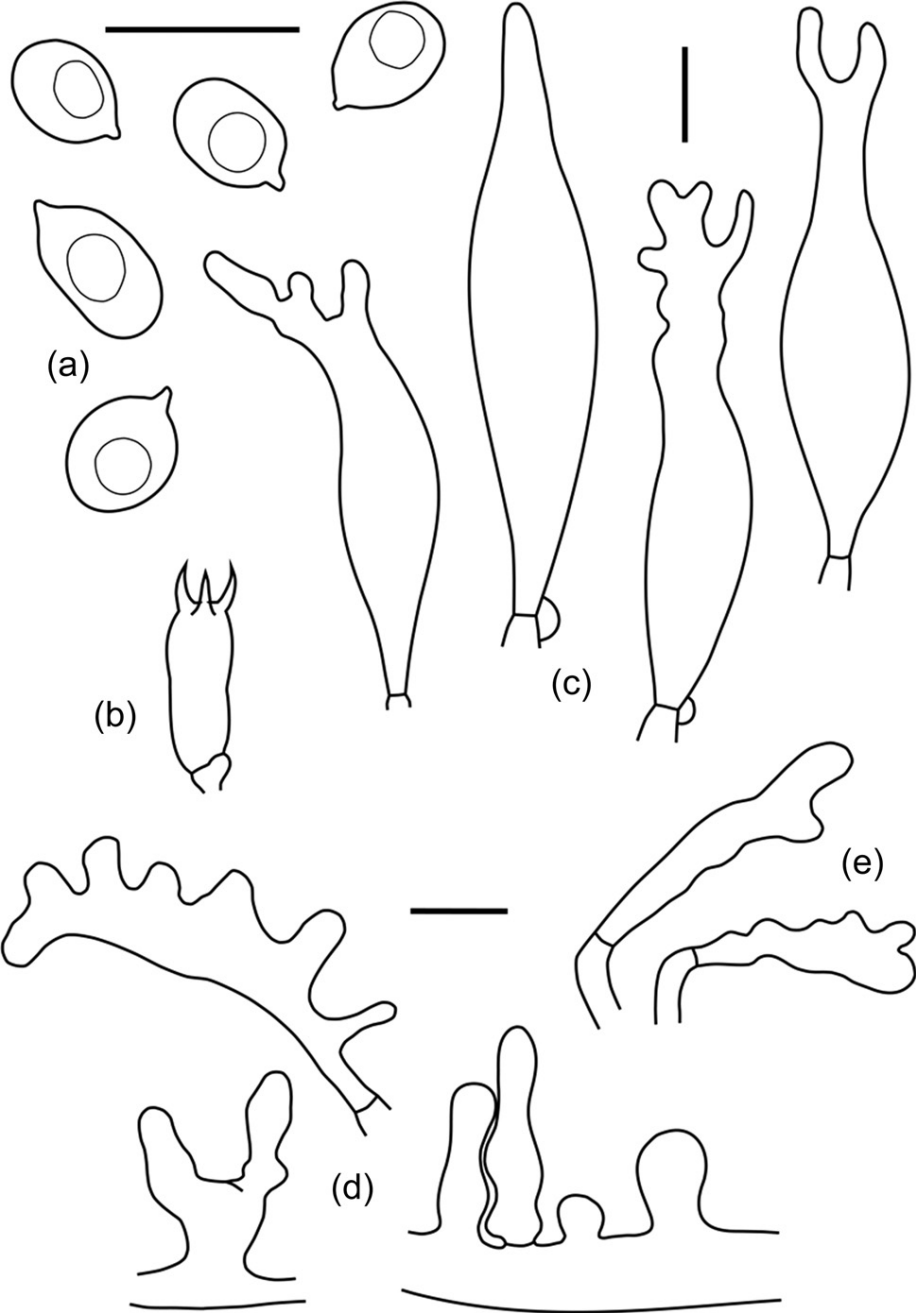
*Filoboletus manipularis* morphotype 1, LE 253914 (G24) – characterized by small basidia, relatively large cheilo-cystidia and much reduced caulocystidia. (a) basidiospores, (b) basidium, (c) cheilocysidia, (d) pileocystidia and (e) caulocystidia. Bars = 10 um.

Spores 6.2–7.5 × 4.4–5.1 µm, *Q* = 1.3–1.6, white, smooth, ellipsoid to broadly ellipsoid, amyloid. Basidia 16.3–18.3 × 6.2–7.5 µm, clamped, narrowly clavate, with sterigmata up to 6 urn long. Cheilocystidia 51.5–73.1 × 6.8–12.9 µm, forming a sterile lamellae edge, lageniform, sub cylindrical or sub-clavate, mostly diverticulate or irregularly branched in the apex, or, rarely, simple lageniform with more or less developed neck. Pleurocystidia not seen. Hyphae of the pileipellis 5–15 urn wide, clamped, with rare excrescences and diverticulate terminal cells. Pileocystidia 25.5–32.9 × 8.2–10.8 µm, abundant, irregularly shaped, lageniform to subclavate, with excrescences. Hyphae of the cortical layer of the stipe 3–8 urn wide, clamped, with rare excrescences and diverticulate terminal cells. Caulocystidia 24.4–38.1 × 6.8–7.4 µm, abundant, irregularly shaped.

*Representatives*. Viet Nam, Dong Nai Prov., Tan Phu Dist., Cat Tiên National Park, on fallen log in lowland semideciduous tropical forest, 4 June 2010, col. O. Morozova, LE 253914; 15 June 2010, LE 253912.

#### Morphology type 2 – Figure 8

Pileus 1.5–6.0 cm in diam., convex, plano-convex to depressed, sometimes with a small papilla in the central depression, hygrophanous, translucently reticulate, smooth, slightly lubricious when moist, whitish with a pinkish tinge, with reddish brown spots in the old basidiomata. Hymenophore tubular, adnate, adnate-emarginate with a decurrent tooth or shortly decurrent. Tubes 2–5 mm long, adnate or adnate-emarginate with a slightly decurrent tooth, in radial rows, with angular pores 0.5–1.5 mm wide. Stipe 20–70 × 0.5–2.5 mm, cylindrical, thickened in the base, hollow, white with a pinkish tinge, brownish toward the base, completely white pruinose.

**Figure 8.**
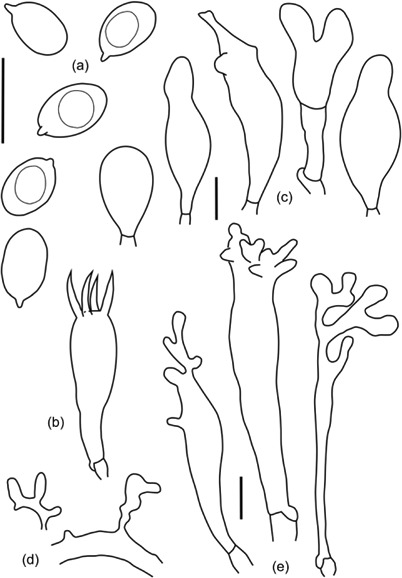
*Filoboletus manipularis* morphotype 2, LE 253916 (G2) – characterized by comparatively large basidia, shorter and simpler cheilocystidia and much reduced pileocystidia. (a) basidiospores, (b) basidium, (c) cheilocysidia, (d) pileocystidia and (e) caulocystidia. Bars = 10 um.

Spores 5.8–8.6 × 4.0–5.5 µm, *Q* = 1.3–1.9, white, smooth, ellipsoid to broadly ellipsoid, amyloid. Basidia 17.4— 35.1 × 7.4–9.7 µm, clamped, narrowly clavate, with sterigmata up to 9.5 urn long. Cheilocystidia 20.5–47.9 × 8.2–10.6 µm, mostly simple lageniform or subcla-vate, rarely bifurcate in the apex. Pleurocystidia not seen. Hyphae of the pileipellis 5–15 urn wide, clamped, with rare excrescences and diverticulate terminal cells, gelatinized. Pileocystidia absent. Hyphae of the cortical layer of the stipe 3–8 um wide, clamped, with rare excrescences and diverticulate terminal cells. Caulocystidia 20.5–138.0 × 6.5–11.0 µm, abundant, irregularly shaped.

*Representatives*. Viet Nam, Dong Nai Prov., Tan Phu Dist., Cat Tiên National Park, on fallen log in lowland semideciduous tropical forest, 3 June 2010, coll. O. Morozova, LE 253917; 4 June 2010, coll. O. Morozova, LE 253915; 8 June 2010, coll. O. Morozova, LE 253916; 21 June 2010, coll. A. Kovalenko, LE 254357.

**Figure 9.**
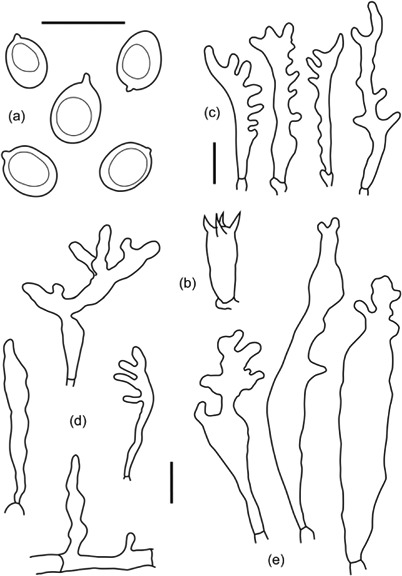
*Filoboletus manipularis* LE 253911 (G25) – characterized by reduced or shrunken cheilocystidia. (a) basidiospores, (b) basidium, (c) cheilocysidia, (d) pileocystidia, (e) caulocystidia. Bars = 10 µm.

Other collections, however, showed more or less intermediate or mixed anatomical features not readily assigned to either morphology, and LE 253911 was notably divergent in having much smaller, narrow and irregularly shaped cheilocystidia, otherwise with basidia and pileocystidia similar to the type 1 morphology but with caulocystidia having the type 2 morphology (Figure 9). Observations that morphological variation was more or less continuous preclude the establishment of formal ‘morphotypes’ for the species based on these observations.

### Phylogenetic analyses

**Figure 10.**
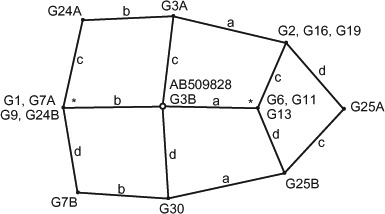
Median-joining network, showing polymorphisms in the internal transcribed spacer (ITS) regions. Lines separating ITS haplotypes represent polymorphisms: (a) adenosine indel at position 195, (b) T/C transition at position 338, (c) adenosine indel at position 538, (d) A/G transition at position 669; * alternate possible positions for AB509828 from Japan.

ITS was sequenced for 13 *F. manipularis* collections, with the ITS1-5.8S-ITS2 region varying from 667 to 669 nt in length. Four sequence polymorphisms were found in the ITS regions from the sequence alignment (Table 4). These were an adenosine indel at position 195 and a T-C transition at position 338 in the ITS1 region, and a second adenosine indel at position 538 and A-G transition at position 669 in the ITS2 region. The 5.8 region and bordering fragments of 28S and 18S were invariant in our samples. Two sequence variants were noted for collections G3, G7, G24 and G25 and unbalanced indels occurred in sequences for G3, G24 and G25, indicating ITS sequence differences including length variants between haplotypes from individual basidiomata in these collections. In total, there were 10 ITS sequence variants noted for all of the collections and median-joining analysis of the polymorphism similarities indicated an interconnected, non-branching genetic relationship among all presumed dikaryon strains (Figure 10). The ITS2 sequence for *F. manipularis* from Japan (Genbank AB509828) was identical to sequences for eight of the Viet Nam collections indicating a close genetic relationship over a broader geographic region than sampled in the current study.

**Table 4.** ITS polymorphisms.

	Polymorphism			
Isolates	195^a^	338^b^	538^c^	669^d^
G2, G16, G19	A	T	A	G
G25A	A	T	A	A
G6, G11, G13	A	T	•	G
G25B	A	T	•	A
Gl, G7A, G9, G24B	•	C	•	G
G7B	•	C	•	A
G24A	•	C	A	G
G3A	•	T	A	G
G30	•	T	•	A
G3B	•	T	•	G
AB509828	–	–	•	G

Notes: ^a^Adenosine indel at position 195 in ITS1.

b T-C transition at position 338 in ITS1.

c Adenosine indel at position 538 in ITS2.

d A-G transition at position 669 in ITS2.

– Missing sequence for ITS1 region.

· nt deletion.

### rpb2

We were unable with *Filoboletus* to successfully apply published protocols to amplify *rpb2* in basidiomycetes (Morehouse et al. 2003; Matheny et al. 2007). Herein we designed primers to amplify a fragment of *rpb2* for *F. manipularis*. These primers succeeded for all of our *Filoboletus* collections, although they did not work for *Mycena*. However, the length of amplified *rpb2* fragments was less than 250 bp, and this gene fragment was not sufficiently informative to further elucidate the genetic structure of the sampled population of *F. manipularis*. Only two sequence polymorphisms were seen in this gene fragment and both were T-C transitions occurring at positions 31 and 57 of the alignment (not shown) discriminating two sequence types. Both *rpb2* sequence variants were observed in Gl6 reflecting sequence differences between the presumed parental monokaryons of this basidioma.

### tef1α

Herein we designed primers to sequence the 5′ region of *tef1α*, which we expected to show a relatively high degree of variation due to the presence of introns. These primers amplified a *tef1α* fragment 843–858 bp in length containing 195 codons interspersed with four introns, the latter varying in length from 50 to 58 bp. After trimming of the 5′ and 3′ ends and deletion of the intron regions, all sequences including *Phyllotopsis* sp. (Genbank DQ059047) and *Mycena plumbea* (GU187729) were uniformly 586 nt in length. Polymorphisms in the *tef1α* exon regions are shown in Table S1 (Supplementary data) and the frequencies of various types of allelic polymorphisms in *F. manipularis* are summarized in Table 5. All 32 polymorphisms observed in the exon regions were single nucleotide polymorphisms (SNPs). The majority of SNPs were nucleotide transitions (27, 84%) with C-T transitions predominant (22, 69%), and with only five nucleotide transversions and no indels. In the intron regions, there were a total of 97 polymorphisms, including 51 (53%) nucleotide transitions, 34 (35%) transversions and 12 indels of 1–4 bp length. C-T transition polymorphisms, as in the exons, predominated in the intron regions with 44 occurrences (45%). Overall, the frequency of polymorphisms in the intron regions (97/207 bp = 46.8%) was approximately nine times higher than in the exons (32/ 586 pb = 5.4%). Three or more nucleotide types were observed at 12 positions in the introns and twice in the exons. The high number of polymorphisms in the introns increases the likelihood of multiple mutations including potential reversals occurring at the same locus; therefore, diversity seen in the exon regions should be phylogenetically more informative.

**Table 5.** Number and relative frequency of allelic polymorphisms in the *tef1α* regions of *F. manipularis*.

Mutation	Exons	Introns	Total
A-G	5 (16%)	7 (7%)	12 (9%)
C-T	22 (69%)	44 (45%)	66 (51%)
Total transitions	27 (84%)	51 (53%)	78 (60%)
A-C	2	9	11
A-T	1	12	13
C-G	2	6	8
G-T	0	7	7
Total transversions	5 (16%)	34 (35%)	39 (30%)
Indels	0	12 (12%)	12 (9%)
Total mutations	32	97	129
Length (nt)	586	207	793

**Figure 11.**
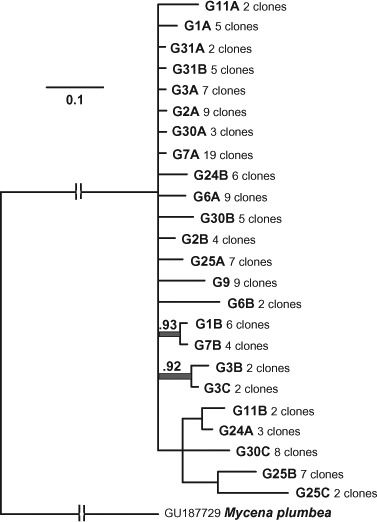
Fifty percent consensus tree from Bayesian analysis of *tef1α* coding region (exons). Posterior probabilities >0.90 indicated above statistically significant branches.

**Figure 12.**
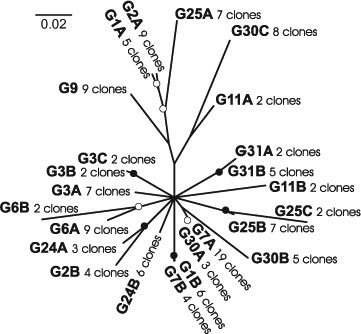
Bayesian analysis of *tef1α* locus including introns. Open circles indicate posterior possibilities (PP) > 0.90; closed circles PP > 0.95; branches collapsed where PP < 0.50.

**Figure 13.**
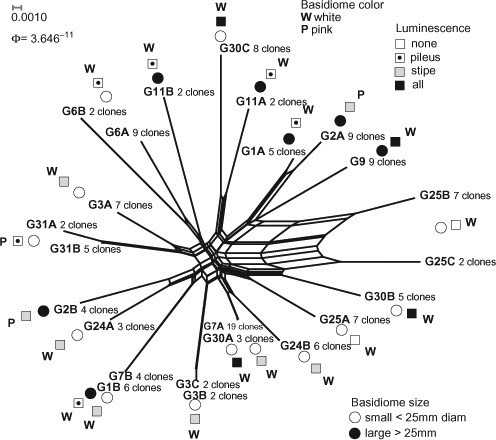
Split decomposition analysis of the *tef1α* locus. Pairwise homoplasy index (PHI) = 3.646^–11^, indicating the presence of recombination across all collections.

The phylogenetic analysis based on *tef1α* exon regions with *Mycena plumbea* (GU187729) as outgroup (Figure 11, http://purl.org/phylo/treebase/phylows/study/TB2:S14862) indicated considerable genetic distance between *Filoboletus* and *Mycena* with limited phylogenetic structure within *F. manipularis*. Analysis of gene diversities showed an unusually low level of heterozygosity among populations from the three different localities within the study area (Fst = −0.023, σ = 0.060, supplementary Table S1) indicating an absence of allelic fixation or inbreeding within localized populations. In the phylogenetic analysis fifteen clone sequence variants were grouped together in a polytomy collapsed at the 50% significance level. Among these, one clone variant (i.e. haplotype representing one presumed parental monokaryon) from G2 had identical exon sequence to one haplotype from each of G7 and G30, and one haplotype from G3 was identical to two haplotypes from G31. Similarly, Gl and G7 had one haplotype with identical exon sequences. However, none of the basidiomata had identical paired haplotypes, indicating a rich assemblage of compatible mating strains in relatively close proximity to one another in these locations. Only two branches within *Filoboletus* were supported at levels higher than 90% and none higher than 95%. One branch joined two haplotypes from G3, and the second joined haplotypes from G1 and G7. However, all three basidiomata had one additional haplotype outside of the supported branches. Significantly, three different haplotype sequences were seen for basidiomata of G3, G25 and G30 (supplementary Table S1). Since DNA for this study was isolated from individual basidiomata, which are presumed to have arisen from conjugation of two compatible monokaryons, this indicates that more than two haploid components could be involved in the development of individual basidiomata, Similar observations were found when the intron regions were included in the analysis (Figure 12, http://purl.org/phylo/treebase/phylows/study/TB2:S14862). Significant branches (>0.95) joined paired haplotypes for G31, G25 and G3, as well as for individual haplotypes from G1 with G7, and G2 with G24. With the exception of G31, the other basidiomata had additional haplotypes arising on unsupported branches (<0.95), again indicating the possibility that more than two component nuclei were involved in the development of individual basidiomata. G7 and G30 each had one haplotype with identical sequence, as did G1 and G7; however, all of these collections originated from the vicinity of Nam Cat Tiên. Recombination patterns were visualized by split decomposition analysis (Figure 13) onto which the morphological characteristics of the basidiomata were summarized. This analysis showed a complex network of relationships and statistically significant recombination within all of *F. manipularis* (Phitest, *Φ* = 3.64E-11). There was no evident pattern of phylogenetic differentiation or correlation of genetic variation with morphological characteristics.

## Discussion

### No apparent phylogenetic basis for observed morphological variation in F. manipularis

Considerable variation was observed in basidioma morphology among the Viet Nam collections, similar to the variation previously reported on a worldwide scale (Corner 1954; Pegler 1986; Manimohan and Leelavathy 1989). Basidiomata at apparent maturity varied in size (0.5–6.0 cm diam.), shape (conical, campanulate or plano-convex to depressed) and color (white to cream, beige or pinkish). Similarly, patterns of luminescence were highly variable, with either the pileus or stipe luminescent, or the entire basidioma luminescent or not. Notably, larger basidiomata were often pink-tinged and appeared to have larger basidia, smaller, essentially inornate cheilocystidia and much larger caulocystidia than smaller basidiomata which tended toward white at maturity. The collection corresponding to G25 (LE 253911) was aberrant in having abnormally small, narrow cheilocystidia with conspicuous lateral invaginations as though collapsing. These are anatomical distinctions that have frequently been used to distinguish species based on morphology. However, we did not find any correlates between any aspect of morphological variation and intraspecific phylogenetic patterns for any of the three gene regions studied. Luminosity also was uncorrelated to any aspect of morphological variation or phylogenetic pattern. Overall, the variation described by Corner (1954) for collections from diverse tropical regions essentially matches our observations. Corner had the opportunity to observe the development over time of fruit bodies of *F. manipularis* collected in northeastern Pakistan. He noted that the fruit bodies were slow in their expansion and remarkably long-lived, producing spores variably for 2–6 days. Significantly, he was able to observe age differences in the appearance of cheilocystidia, caulocystidia and pileocystidia, with cheilocystidia and pileocystidia collapsing on expansion of the pileus. This provides a likely explanation for the aberrant and apparently senescent cheilocystidia observed in LE 253911 in our study. Corner (1954) also noted a progression of color changes over the life of the basidiomata, and concluded that pink and reddish discoloration in age might be attributable to the presence of molds (e.g. Põldmaa 2011).

Conceivably, genetic variation between sexually compatible monokaryons could contribute to the morphological variation observed. However, environmental factors such as climate, local weather patterns or substrate suitability, and developmental factors such as age in particular are very likely to be significant determinants of basidioma morphology and luminosity. In the current study anatomical observations may have been significantly impacted by varying conditions of drying or preservation since the collections had to be shipped and were not immediately available for microscopic observation. All these factors advise caution in interpreting morphological and anatomical characters to differentiate and define species in *Filoboletus*.

### Filoboletus manipularis is a single phylogenetic species as currently recognized

Only four polymorphisms were found in a 667–669 bp sequence fragment of ribosomal DNA which included the ITS 1 and 2 regions. This represented a level of ITS variability of 0.6%, which is well within the generally accepted level of intraspecific ITS variation of approximately 0–3% (Basidiomycota average 3.3%, Nilsson et al. 2008). The only published sequence for *F. manipularis* (Genbank AB502898 from southern Japan) also fell within this range of variation having an identical ITS2 sequence to 6 of the 10 Vietnamese collections. Unique haplotype sequences were observed in G7 and G30. All other collections shared identical haplotype sequences in varying combinations, indicating a complexly interconnected relationship among all of the collections. In contrast, ‘*Filoboletus* aff. *manipularis*’ (AB509539), when compared to *F. manipularis* (AB502898), had 24 polymorphisms within the ITS2 region (209 nt's) including 9 indels of 1–2 bp, thus representing a frequency of ITS variation of 11.5% and indicative of a separate species. Our observations of an approximately 250 bp fragment of *rpb2* also indicated a close, interconnected phylogenetic relationship for the collections studied, with only three T-C nucleotide transitions differentiating the collections. Identical haplotype sequences also were observed from morphologically distinct basidiomata in analyses of the *tef1α* locus, reinforcing the conclusion that the Viet Nam collections represent a single species. The estimated coefficient of inbreeding was unusually low (Fst = −0.023), indicating a lack of allelic fixation within local populations sampled in this study. Conclusively, the analysis of split frequencies based on the *tef1α* sequence data found statistically significant recombination among all of the *F. manipularis* collections and no correlation between genetic relationships and morphological characteristics.

### Could basidiomata be ‘polykaryotic’ or chimeric?

The detection of three different *tef1α* haplotype sequences from single basidiomata of G3, G25 and G30 indicates a possibility that more than two monokaryons contributed to the formation of individual basidiomata. A high fidelity ‘proofreading’ polymerase was used for PCR after cloning; nevertheless, 12 unique (‘singleton’) clone sequences were excluded from the phylogenetic analyses to eliminate the possibility of cloning errors caused by the incorrect incorporation of nucleotides. Assuming the occurrence of mutations is random, the possibility of any two sequences from one basidioma having the same mutation can be approximated using the calculation provided by Vydryakova et al. (2011) based on the ‘Birthday Paradox'. For G3, G25 and G30, there are 15–17 polymorphic regions and the possibility of random variation explaining two occurrences of any one transition polymorphism is approximately *P* ∼ 0.148 and of three occurrences *P* ∼ 0.022, or half these probabilities for a transversion mutation. For G3 the two 2-clone sequences differ by a single base, a G-C transversion, which could statistically (*P* ∼ 0.074) be attributed to cloning error, that if correct would leave two haplotypes represented by 7 and 4 clones, respectively, and differing by 17 distinct polymorphisms. For G25, there are 28 total polymorphisms differentiating the 2-clone sequence and the two 7-clone sequences. However, the 2-clone sequence has only two unique polymorphisms, otherwise having one or the other polymorphisms of either of the two 7-clone sequences, suggesting it could be a recombinant derived from the 7-clone sequences with one crossover occurring between positions 513–579 of the alignment (Table 6). However, the probability of occurrence of the two unique polymorphisms in the 2-clone sequence, a C-T transition and an A-T transversion, is P-0.011. Therefore, it would appear more likely that the 2-clone sequence is a unique third haplotype in G25. For G30 there are 29 polymorphisms involving the 3-, 5- and 8-clone sequences (Table 7). The 3-clone sequence in G30 is unique in four polymorphisms, including a 4-base substitution, two C-T transitions and a 2-base deletion that statistically cannot be attributed to cloning error leaving the additional possibility of three parental monokaryons contributing to the G30 basidioma. We had a closer look at the 12 unique clone sequences ('singleton’ clones) excluded from the previous analyses to see if they showed significant patterns of variation. Four clones had unique polymorphisms that could have resulted from PCR replication errors propagated by cloning. All polymorphic loci in the remaining eight singleton clone sequences had the same polymorphisms as one or more other clones. Many clones had polymorphisms in common with one or another of the dominant clones suggestive of recent recombination involving the dominant clones. In G3, the pattern of polymorphisms in all four singleton clones could be explained by one or two crossover events occurring within a span of about 700 nts (Table 8). However, the singleton clone in G6 (Table 9) would require eight crossover events to have been derived by recombination from the dominant clones, with which it shares 21 polymorphic values in common with one or the other dominant clone. Clearly this level of crossing-over reflects a long evolutionary history of recombination events. A more complicated pattern of relationships is evident in the clones from G7 (Table 10). Singleton clones D and E have two and five unique polymorphisms, respectively, and three of these polymorphic loci are also polymorphic between the dominant clones. In addition, polymorphisms occurring in differing combinations of singleton clones and not appearing in the dominant clones occur at ten polymorphic loci. Additional singleton clones were observed from basidiomes G30 and G31 (not shown) that were similarly indicative of haplotypes different from the dominant haplotypes. Overall, the pattern of distribution of shared polymorphisms suggests the occurrence in individual basidiomata of multiple haploid components that have arisen following a long phylogenetic history of mutation and recombination.

**Table 6.**
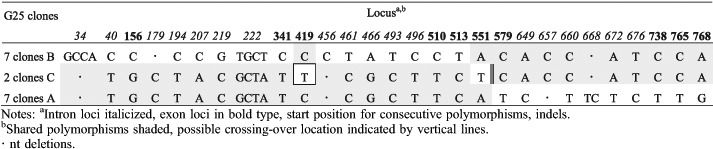
*tef1α* polymorphisms in G25 clones.

**Table 7.**
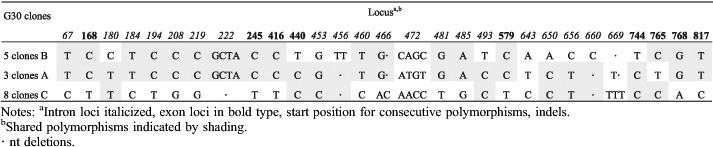
*tef1α* polymorphisms in G30 clones.

**Table 8.**
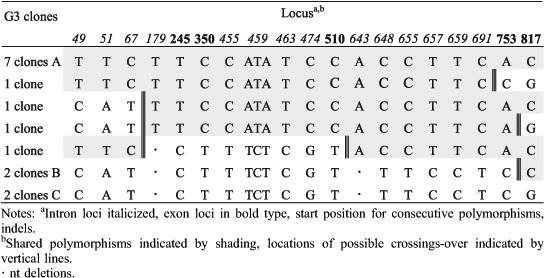
*tef1α* polymorphisms in G3 clones.

**Table 9.**
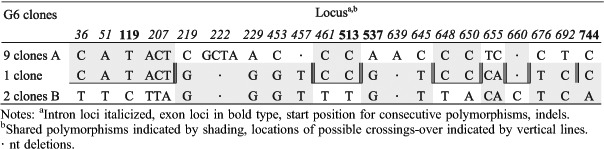
*tef1α* polymorphisms in G6 clones.

**Table 10.**
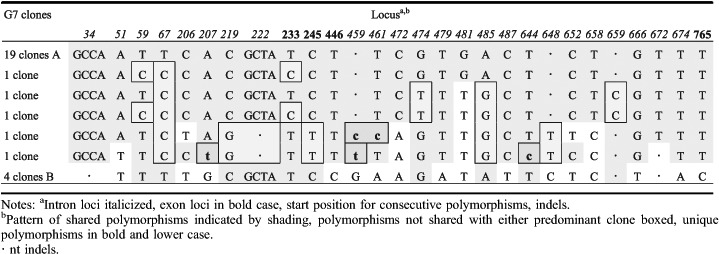
*tef1α* polymorphisms in G7 clones.

In planning this study, we believed we would find, at most, two different haploid sequences representing the two parental monokaryons contributing to formation of individual basidioma. However, more than two haploid sequences were found among clones from some basidiomata which could not be attributed to cloning errors arising from nucleotide misincorporation. Highly variable products of recombination could occur in the generative tissues which could be propagated by PCR, and these recombinants could be common if a significant portion of the genetic material is translocated from structural and generative tissues and devoted to sexual recombination. Patterns suggestive of recombination were seen for some of the ‘singleton’ clone sequences, as well as for two identical clones sequenced from G25. However, in most cases the pattern of recombination was overly complex to be attributable to recent recombination within a single basidioma, requiring frequent and close-spaced crossing-over. Finally, in G30 there were three different and statistically significant clone sequences suggestive of three parental monokaryons, and multiple haploid sequences were also observed in G7 and G31 that could not be explained by recent meiotic recombination.

Vydryakova et al. (2011) reported similar observations for the ITS locus in *Neonothopanus nambi* (Speg.) R.H. Petersen & Krisai, also suggestive of a ‘polykaryotic’ condition in basidiomata of this species. However, since ITS is a multicopy genetic locus these authors recognized the possibility that variability in the ITS could potentially be attributed to the existence of multiple copies of the rRNA cistron evolving independently by being separately located in unlinked regions of the genome. *Tefla* has generally been considered to be a single-copy gene. However, there is increasing evidence for the occurrence of paralogous copies of the *tef1α* gene in fungi (Aguileta et al. 2008). A search for the *tef1α* gene in complete genomes of Agaricomycotina in Mycocosm using search term KOG0052 indicated that *tef1α* is a single-copy gene in approximately 90% of the agaric species covered (http://genome.jgi.doe.gov/programs/fungi/index.jsf, Grigoriev et al. 2012). Multiple *tef1α* copies occurred in only 9 of the 94 genomes annotated to date, none of which were closely related to *Filoboletus*, and multiple copies were not seen in *Tricholoma matsutake*, which was the only representative of the Tricholomatoid clade (Matheny et al. 2006). The lack of full-genome coverage for the Mycenaceae leaves uncertainty concerning the probable copy state for the gene in *Filoboletus*. However, if multiple copies of *tef1α* were amplified in the current study we would expect to see patterns of sequence divergence for the paralogous copies. Instead, the variation appears more or less random and continuous and split decomposition analysis of recombination patterns indicated a statistically significant level of recombination across all haplotype sequences. Therefore, we conclude that a single locus for the *tef1α* gene was amplified in this study. An additional concern for the current study is the possibility of PCR-mediated recombination contributing to the sequence variation observed for *tef1α*. Chimeras are produced when a prematurely terminated amplicon reanneals to foreign DNA and is copied to completion in a subsequent amplification. The high-fidelity polymerase employed in this study requires a short extension period which has been associated with PCR-mediated recombination. However, this polymerase utilizes a low starting template concentration which should compensate to decrease the likelihood of chimeras being produced (Lahr and Katz 2009). It is difficult to distinguish chimeric sequences from sequence variants resulting from meiotic recombination. However, most of the haplotype sequences in the current study are distinguished by SNPs and indels in addition to crossovers that could have resulted from PCR-mediated recombination or meiotic recombination. Break points occurred at the exactly the same locations in multiple haplotypes and most haplotypes had a pattern of frequent and close-spaced crossovers unlikely to be seen in PCR-mediated chimeras. Instead the pattern of variation observed in this study is indicative of a long history of recombination and inherited mutations and provides compelling evidence for the occurrence of three or more parental monokaryons contributing to basidioma development. Two mechanisms that might result in multinucleate basidiomata are discussed below.

### Multinucleate basidiomata develop directly from trikaryotic or polykaryotic mycelium in multinucleate basidiomycetes

Germinating basidiospores and mitotically generated oidia readily fuse with compatible mycelia to form the dikaryon mycelial phase that dominates the basidiomycete life cycle. However, the nuclear condition of the dikaryon phase is not stable, and di-mon mating – dikaryotization of a monokaryon following mating with an existing dikaryon originally described by Buller (1930) – may occur frequently in nature. Stable trikaryons, following di-mon matings, have been observed in pairings between heterokaryons and homokaryons in basidiomycetes with multinucleate cells (James et al. 2009). As many as 30% or more of basidiomycetes may have multinucleate cells ('cenocyty', Boidin 1971). Therefore, if tri-genomic mycelia undergo recombination through completion of the sexual cycle (Kimura and Kadoya 1962), multinucleate basidiomata may occur relatively frequently in basidiomycetes with multinucleate mycelium. Koltin and Raper (1968) also found evidence for the occurrence of a diploid mycelium in *Schizophyllum* capable of anastomosing with haploid mycelium to establish ‘haploid-diploid dikaryons’ in which the diploid nucleus appeared unstable resulting in a mosaic of two different ‘haploidhaploid dikaryons’ in the resulting basidiomata. However, in our observations clamp connections were formed regularly in *F. manipularis* perhaps indicating against the possibility of a polykaryotic state being maintained to formation of basidiomata.

### Adjacent mycelium representing two different dikaryons conjoin to form a mycelial knot in the initial stage of basidiome formation with resultant chimeric basidiomata

Following dikaryotization, hyphae readily fuse to allow active translocation of limiting nutrients throughout a mycelium (Olsson and Gray 1998). Nuclear replacement following anastomosis is unidirectional toward the hyphal tips (Kűes 2000) and following sequential anastomoses originally separate clones may act as a single individual. Thus a mycelium may be a chimera of genetically different mycelial parts benefiting mutually in exploiting distant food sources by translocation of nutrients throughout the mycelial network. In addition, somatic recombination occurs at low frequencies in both trikaryon and dikaryon mycelium (Shalev et al. 1972; Korhonen and Hintikka 1974; Frankel 1979; Anderson and Kohn 2007) and multiple nuclear haplotypes reassorted from somatically incompatible mycelia have been found residing in the same mycelium (Johannesson and Stenlid 2004). Somatic incompatibilities do not necessarily result in the effective segregation of new-formed dikaryon pairings and a strictly compatible dikaryon condition is not always required for fruit body formation in basidiomycetes. Incompatibility at the B mating-type locus, for example, does not preclude fruit body formation and basidiomata can develop readily from common B heterokaryons (Casselton and Kűes 1994; Kűes 2000). Fruit body formation in the basidiomycetes is not necessarily monocentric and hyphal knots representing the initial stage in fruit body formation commonly originate from more than one generative hypha, with branches from neighboring hyphae anastomosing to form the basidioma initial (Kűes 2000, observations in *Coprinus cinereus, = Coprinopsis cinerea*, Psathyrellaceae). Since only the young hyphae at the margin of the colonies can produce basidiomata (Kűes 2000), it is conceivable that adjacent mycelia with different nuclear types could come in contact at the time of fruiting. Genetic differences potentiate rejection and acceptance mechanisms; however, interaction outcomes are dependent on both genes and context and the ability of nonself genomes to conjoin may be dependent on levels of oxidative stress (Rayner 1996). Similarly the frequency of cenocytic mycelium and multinucleate cells is increased under conditions of insufficient aeration (Boidin 1971). The formation and tolerance of chimeric mycelial communities through hyphal anastomoses advantage the supply of nutrition during fruit body development. In the absence of a strict nonself recognition mechanism, anastomoses between adjacent, genetically different dikaryons in the mycelial knot stage of basidioma formation could result in chimeric basidiomata. Seamless chimeric basidiomata between genera have been recorded in two different situations. Kemp (1977) reported chimeric basidiomata jointly formed by *Parasola misera* (P. Karst.) Redhead, Vilgalys & Hopple and *Coprinellus pellucidus* (P. Karst.) Redhead, Vilgalys & Moncalvo (both in Psathyrellaceae and reported as *‘Coprinus’* species) that had colonized the same dung sample. The mycelia of the two species were clearly not antagonistic and he was able to distinguish the mycelium of the two species in the stipe as well as tetrads of spores of both species in the gills. Other chimeric basidiomata involve the mycoparasitic genus *Squamanita*. Mushroom species such as *Squamanita contortipes* (A.H. Sm. & D.E. Stuntz) Heinem. & Thoen and *Squamanita paradoxa* (A. H. Sm. & Singer) Bas, form chimeric basidiomata with their agaric hosts, a *Galerina* for *S. contortipes*, and a *Cystoderma* with *S. paradoxa* (Redhead et al. 1994; Matheny and Griffith 2010), as do other *Squamanita* species with their hosts (Reid 1983; Harmaja 1988; Nagasawa et al. 1990).

In the case envisaged for *Filoboletus*, the apparent nonselfish collaboration of genetically different nuclei in the translocation of nutrients may serve to enhance the vigor of a chimeric mycelium allowing adaptation to changing or unfavorable environmental conditions. The existence of polykaryotic basidiomata also would be significant in reducing barriers to genetic recombination possibly impacting the rate of evolution of new species. Unfortunately, only a single monospore culture of *F. manipularis* was recovered from the field, and other material was not available in this study to determine the nuclear status of the mycelium, or to study nonself confrontation reactions in mycelial contact zones between different dikaryons. In the current study spores from different basidiomata of *Filoboletus* occurring in proximity could have contaminated our samples and the possibility of cloning errors and multiple gene copies cannot be completely discounted. Most significantly there is evidence from full genome sequences for the occurrence of multiple copies of the *tef1α* gene in approximately 10% of basidiomycete species with no information on the possible occurrence of polyploidy or aneuploidy in *Filoboletus* or close relatives. Therefore, additional research is necessary to confirm a perhaps common phenomenon of three or more component nuclei representing multiple parental monokaryons contributing to the development of individual basidiomata.
